# Store-and-Forward Images in Teledermatology: Narrative Literature Review

**DOI:** 10.2196/37517

**Published:** 2022-07-18

**Authors:** Simon W Jiang, Michael Seth Flynn, Jeffery T Kwock, Matilda W Nicholas

**Affiliations:** 1 Department of Dermatology Duke University School of Medicine Durham, NC United States

**Keywords:** store-and-forward, patient, clinician, telehealth, COVID-19, teledermatology, image, photograph, asynchronous, practice, outcome

## Abstract

**Background:**

Store-and-forward (SAF) teledermatology uses electronically stored information, including patient photographs and demographic information, for clinical decision-making asynchronous to the patient encounter. The integration of SAF teledermatology into clinical practice has been increasing in recent years, especially during the COVID-19 pandemic. Despite this growth, data regarding the outcomes of SAF teledermatology are limited. A key distinction among current literature involves comparing the quality and utility of images obtained by patients and trained clinicians, as these metrics may vary by the clinical expertise of the photographer.

**Objective:**

This narrative literature review aimed to characterize the outcomes of SAF teledermatology through the lens of patient- versus clinician-initiated photography and highlight important future directions for and challenges of the field.

**Methods:**

A literature search of peer-reviewed research was performed between February and April 2021. Key search terms included *patient-initiated*, *patient-submitted*, *clinician-initiated*, *clinician-submitted*, *store-and-forward*, *asynchronous*, *remote*, *image*, *photograph*, and *teledermatology*. Only studies published after 2001 in English were included. In total, 47 studies were identified from the PubMed electronic database and Google Scholar after omitting duplicate articles.

**Results:**

Image quality and diagnostic concordance are generally lower and more variable with patient-submitted images, which may impact their decision-making utility. SAF teledermatology can improve the efficiency of and access to care when photographs are taken by either clinicians or patients. The clinical outcomes of clinician-submitted images are comparable to those of in-person visits in the few studies that have investigated these outcomes. Coinciding with the onset of the COVID-19 pandemic, asynchronous teledermatology helped minimize unnecessary in-person visits in the outpatient setting, as many uncomplicated conditions could be adequately managed remotely via images captured by patients and referring clinicians. For the inpatient setting, SAF teledermatology minimized unnecessary contact during dermatology consultations, although current studies are limited by the heterogeneity of their outcomes.

**Conclusions:**

In general, photographs taken by trained clinicians are higher quality and have better and more relevant diagnostic and clinical outcomes. SAF teledermatology helped clinicians avoid unnecessary physical contact with patients in the outpatient and inpatient settings during the COVID-19 pandemic. Asynchronous teledermatology will likely play a greater role in the future as SAF images become integrated into synchronous teledermatology workflows. However, the obstacles summarized in this review should be addressed before its widespread implementation into clinical practice.

## Introduction

The role of telecommunications in clinical dermatology (teledermatology) is continually expanding as technology becomes an inextricable component of medical practice. The COVID-19 pandemic has driven it to the forefront of many dermatology practices around the world, often with rapid implementation spurred more by necessity than methodology. Teledermatology can be classified by the temporal relationship between the clinician’s decision-making and the patient encounter. Synchronous teledermatology takes the form of web-based, real-time patient visits and is outside the scope of this review. Asynchronous, or store-and-forward (SAF), teledermatology uses electronically stored information, such as patient photographs and demographic information, for medical decision-making.

Data on SAF teledermatology vary considerably depending on how studies are structured. A key element of experimental setup is whether the SAF images are acquired by a trained clinician or the patient. Intuitively, variation in the quality and utility of patient-submitted images is to be expected. These characteristics may depend on whether a patient possesses a high-quality camera, their understanding of clinical photography, and their access to assistance with taking photographs—elements that are more readily available in the clinical setting. Characterizing the differences in SAF images submitted by clinicians versus patients is crucial as more health care systems integrate teledermatology consultation programs into clinical practice. Given the lack of comprehensive articles regarding this distinction, this review will explore the outcomes, consider the impacts of COVID-19, and highlight the future directions of asynchronous teledermatology based on whether photographs are taken by clinicians or patients.

## Methods

A narrative review of peer-reviewed literature was performed between February and April 2021 to identify articles pertaining to SAF teledermatology with clinician- and patient-initiated images. Key search terms included *patient-initiated*, *patient-submitted*, *clinician-initiated*, *clinician-submitted*, *store-and-forward*, *asynchronous*, *remote*, *image*, *photograph*, and *teledermatology*. The study designs of the identified literature included a meta-analysis, systematic reviews, randomized controlled trials, and observational studies.

Only studies published after 2001 were included in the search criteria, although a substantial number of articles related to SAF teledermatology were published in the past decade. In total, 47 studies were selected from the PubMed electronic database and Google Scholar after omitting duplicate articles. Inclusion criteria consisted of articles that primarily examined the clinical aspects of SAF teledermatology, such as diagnosis, waiting intervals, change in management, clinical outcomes, and image quality. Survey studies and observational reports were also included if they primarily focused on the use of SAF teledermatology in patient care. Studies that investigated synchronous but not asynchronous teledermatology, focused on SAF teledermatology outside of patient care (eg, economic analyses), and were not available in English were excluded. In total, 2 independent researchers with knowledge of study interpretation and literature review performed separate screenings of the literature and validated their search results. Several studies in this review met the exclusion criteria but were included as discussion points rather than for result interpretation.

## Results

### Image Quality

The evaluation of a photographed skin condition can be heavily influenced by its image quality. Several studies that assessed images taken by trained clinicians found that those deemed of low or poor quality ranged from approximately 5% to 20% [1-4]. In contrast, the quality of patient-initiated images is more variable. One study of patients who submitted smartphone images of their skin lesions to dermatologists found that around half took their own photographs [5]. The authors excluded nearly 10% of the images from assessment due to poor image quality [5]. Given that this study population consisted of university students, the number of poor-quality images could be much higher in populations with lower technological proficiency or those without assistance in capturing photographs [5]. Other studies with similar experimental setups have observed that low-quality images comprised approximately 10% to 40% of all patient-submitted photographs [6-8]. Though data indicate that clinician-initiated images are generally higher quality than patient-initiated images, standardizing the photography of skin conditions may be useful for teledermatologists receiving primary care referrals and direct patient messages. For instance, tools in the electronic health record (EHR) could remind patients and referring clinicians to provide images with the appropriate lighting, field of view, and focus [9].

### Diagnostic Agreement and Accuracy

Interrater agreement refers to the degree to which the responses of 2 or more raters are similar [10]. When responses pertain to the diagnosis of a disease, it is called diagnostic agreement or concordance, which can be reported as exact agreement between evaluators or as the sum of exact agreement and weighted partial agreement of categorically similar diagnoses [11]. The diagnostic concordance rates between teledermatologists evaluating SAF images and dermatologists seeing patients face-to-face (FTF) range from approximately 60% to 90% for the studies included in this review [2-4,12,13] (Table 1). One study found that the agreement between 2 dermatologists who evaluated images remotely was 68% compared to 88% concordance when these same dermatologists evaluated patients at a FTF visit [3] (Table 1). A recent meta-analysis found that FTF diagnostic concordance rates are significantly higher than remote concordance rates, although the study did not stratify by whether the SAF images were generated by clinicians or patients [14]. Notably, 3 of the 6 studies included in the meta-analysis were published prior to 2000, indicating a need for more up-to-date research [14]. Another consideration that may impact diagnostic concordance is the training and practice setting of the referring clinician. Pasadyn et al [15] identified that diagnostic agreement was highest (50%) between teledermatologists and physicians referring from office visits, compared to teledermatologists and nurse practitioners, physician assistants, or physicians referring from walk-in clinics (around 30%; Table 1).

Recent evidence suggests that the diagnostic utility of SAF images depends on lesion type. Warshaw et al [1] found that diagnostic concordance between SAF images evaluated by a teledermatologist and those same conditions examined in-person were higher for pigmented lesions than nonpigmented lesions. Interestingly, they observed that concordance between management recommendations made by a teledermatologist and an in-person dermatologist was lower when evaluating pigmented lesions, which may be in part due to the option to write in answers for decision-making [1] (Table 1).

**Table 1:** Diagnostic outcomes for store-and-forward teledermatology. The results are reported as percentage exact agreement or percentage exact and partial agreement with a 95% CI.

Type		Setting	Sample	Outcome	Reference
**Clinician-initiated**					
	Observational	Single-center study in the United States (Minnesota)	2152 patients	52.8% to 93.9% diagnostic agreement for pigmented lesions, 47.7% to 87.3% diagnostic agreement for nonpigmented lesions, 66.7% to 79.8% management agreement for pigmented lesions, and 72% to 86.1% management agreement for nonpigmented lesions	Warshaw et al [1]
	Observational	Single-center study in the United States (Wisconsin)	135 children	82% agreement between TD^a^ and FTF^b^ diagnosis (95% CI 73%-88%)	Heffner et al [2]
	Observational	Web-based app in Sweden	40 adults	68% interobserver agreement for TD diagnosis (95% CI 51%-81%), and 88% interobserver agreement for FTF diagnosis (95% CI 73%-96%)	Börve et al [3]
	Systematic review	N/A^c^	25 studies	62% to 89% agreement between TD and FTF diagnosis	Rat et al [4]
	Observational	Single-center study in Austria	18 adults	89% exact agreement between TD and FTF diagnosis	Massone et al [12]
	Observational	Single-center study in the United States (California)	86 adults	82% agreement between TD and FTF diagnosis (95% CI 73%-89%)	Lamel et al [13]
	Observational	Single-center study in the United States (Ohio)	318 clinic visits	MD^d^/DO^e^: 50% exact diagnostic agreement between TD and office visit, and 29.8% exact diagnostic agreement between TD and walk-in clinic; NP^f^/PA^g^: 33.8% exact diagnostic agreement between TD and office visit, and 34% exact diagnostic agreement between TD and walk-in clinic; diagnostic agreement was higher for MD/DO office visits than MD/DO walk-in clinics (*P*=.021), NP/PA office visits (*P*=.035), and NP/PA walk-in clinics (*P*=.022)	Pasadyn et al [15]
**Patient-initiated**					
	Observational	Single-center study in Australia	55 adults	69% exact agreement between TD and FTF diagnosis	Boyce et al [5]
	Observational	Single-center study in Austria	263 adults	49% exact agreement between TD and FTF diagnosis^h^; significant correlation between correct diagnosis and image quality (*P*<.001)	Weingast et al [8]
	Randomized controlled trial	Single-center study in the United States (Pennsylvania)	40 children	83% agreement between TD and FTF diagnosis (95% CI 71%-94%)	O’Conner et al [16]
	Observational	Single-center study in the Netherlands	96 adults	41% exact agreement between TD and FTF diagnosis	Eminović et al [17]

^a^TD: teledermatology.

^b^FTF: face-to-face.

^c^N/A: not applicable.

^d^MD: Doctor of Medicine.

^e^DO: Doctor of Osteopathic Medicine.

^f^NP: nurse practitioner.

^g^PA: physician assistant.

^h^Includes cases that dermatologists indicated as not possible to diagnose.

Compared to clinician-initiated images, patient-initiated images have diagnostic concordance rates that are lower and more variable. Several studies indicate that diagnostic concordance rates between dermatologists evaluating patient-generated SAF images and dermatologists evaluating patients at a FTF visit range from approximately 40% to 80% [5,8,16,17] (Table 1). One of these studies used patient-acquired dermoscopic images to monitor atypical nevi, indicating that patients may be able to acquire highly useful images when provided adequate instructions [18]. Importantly, Weingast et al [8] observed that diagnostic agreement significantly correlated with image quality. The current literature on patient-initiated images is limited by the generalizability of the patient cohorts due to the dearth of studies. For instance, 2 studies had mean ages of 36 and 39 years, whereas 2 other studies were conducted in the pediatric setting in which parents took photographs of their children [6,8,16,17] (Table 1). Such groups may have more technological proficiency than the average adult dermatology patient, which could skew these studies toward higher estimates of image quality than in actual practice.

Diagnoses based on SAF teledermatology images can also be compared to histopathological reports, referred to here as diagnostic accuracy. A recent study found that the diagnostic accuracy of clinician-initiated images was higher for malignant diagnoses such as melanomas and nonmelanoma skin cancers than benign diagnoses [19]. However, there was higher interobserver concordance between teledermatologists and in-person dermatologists when they examined benign diagnoses [19]. To date, there are no studies that evaluate the diagnostic accuracy of patient-initiated images.

In summary, the rates of diagnostic concordance between SAF teledermatology and FTF clinic visits are higher and less variable when skin conditions are photographed by clinicians. Agreement can be impacted by several factors, including the practice type of the referring clinician, type of lesion being photographed, and image quality. Studies that evaluate the diagnostic outcomes of patient-initiated SAF images in a real-life setting are needed.

### Change in Condition, Waiting Interval, and Other Clinical Outcomes

Aside from diagnostic concordance, other outcomes that are relevant to SAF teledermatology may include change in a skin condition and waiting interval between consultation and appointment, among several others. For images taken by clinicians, outcomes appear to be generally similar between SAF teledermatology and FTF visits (Table 2). A prospective study by Pak et al [20] found that the clinical outcomes of asynchronous consults and conventional in-person visits were not significantly different based on a 3-point scale rated by a dermatologist, with 65% and 64% of clinical outcomes being rated as improved in the usual care group and the teledermatology group, respectively (Table 2). Whited et al [21] conducted a randomized controlled trial comparing outcomes at 3-month and 9-month timepoints after primary care physician (PCP) referral [21]. They found no significant difference in the quality-of-life metric Skindex-16 at these timepoints between patients randomized to SAF or conventional consultations [21] (Table 2).

Several studies investigated the waiting intervals between initial consultation and subsequent clinic visit for clinician-initiated SAF teledermatology and traditional referral systems. They all found that SAF teledermatology significantly reduced the time between referral and clinic visit [22-24]. One study observed that SAF teledermatology referral not only reduced the time until consultation completion but also the time to biopsy and surgery for applicable patients [23]. The benefit of this reduced waiting interval may have contributed to the adoption of electronic dermatology referrals over traditional letter referrals in many health care systems.

The clinical outcomes of patient-submitted images are mostly descriptive in nature. Hubiche et al [6] found that SAF images taken prior to in-person evaluation changed treatment decisions in 36% of patients (Table 2). Notably, skin lesions had changed in 87% of patients at in-person evaluation compared to prior photographs [6]. This may indicate that patient images provide useful information for tracking the evolution of a skin condition. However, it is possible that the additional information may in fact obfuscate the correct diagnosis and management, given that the study did not examine any further outcomes [6] (Table 2). Regarding waiting interval, one study that implemented a direct-care teledermatology program reported an average time of <1 day from patient concern to teledermatologist assessment [25]. Eminović et al [17] used SAF teledermatology as a triage tool based on patient-submitted images collected by their PCPs. The authors found that 23% of patients could have avoided FTF appointments, as determined by a panel of 3 dermatologists [17] (Table 2). Notably, there is a lack of data comparing the outcomes of SAF teledermatology based on patient-submitted images to other forms of care, such as FTF care and clinician-initiated teledermatology. As more health care systems allow patients to directly send photographs to their dermatologists, elucidating these outcomes becomes increasingly important.

In summary, outcomes such as change in condition and the quality of life between clinician-initiated SAF teledermatology and FTF care are not significantly different. However, there are a limited number of studies that examine clinically relevant outcomes, and more research is needed. Waiting intervals between SAF referral and FTF appointment are significantly decreased compared to conventional referral systems. Patient-initiated images could supplement decision-making but lack comparable outcomes to other forms of dermatologic care.

**Table 2:** Clinical outcomes of store-and-forward teledermatology.

Type		Setting	Sample	Outcome	Reference
**Clinician-initiated**					
	Observational	2-center study in the United States (Texas)	508 adults	No significant difference between TD^a^ (65% improved, 32% unchanged, and 3% worsened) and FTF^b^ care (64% improved, 33% unchanged, and 4% worsened) as rated by a 3-point clinical course scale (*P*=.57)	Pak et al [20]
	Randomized controlled trial	2-center study in the United States (Missouri and Minnesota)	326 adults	No significant difference between TD and FTF care as evaluated by Skindex-16 at 3 (*P*=.66) and 9 (*P*=.39) months	Whited et al [21]
	Observational	Multicenter study in Spain	2009 adults	51.2% of patients with TD consults not referred to FTF clinic; waiting interval to clinic appointment was 12.31 (95% CI 8.22-16.40) days for TD referral and 88.62 (95% CI 38.42-138.82) days for traditional letter referral system	Moreno-Ramirez et al [22]
	Observational	Single-center study in the United States (California)	149 adults	Mean time interval for TD versus conventional referral was 4 versus 48 days (*P*<.0001) for initial consult completion; 38 versus 57 days (*P*=.034) for time to biopsy; and 104 versus 125 days (*P*=.006) for time to surgery	Hsiao et al [23]
	Randomized controlled trial	Single-center study in France	103 patients	Waiting interval to clinic was 4 days for TD referral and 40 days for conventional letter referral system (*P*<.01)	Piette et al [24]
**Patient-initiated**					
	Observational	Single-center study in France	162 adults and children	Photographs of a skin lesion taken before a clinic visit changed treatment decisions in 36% of patients	Hubiche et al [6]
	Observational	Single-center study in the Netherlands	105 adults and children	23% of patients could have avoided FTF care, as determined by 3 dermatologists	Eminović et al [17]
	Observational	Single-center study in the United States (California)	38 adults	Average time from patient concern to consultation was 0.8 (SD 1) days, and 75% of concerns could be managed remotely	Pathipati et al [25]

^a^TD: teledermatology.

^b^FTF: face-to-face.

### Access to Care

**Figure 1:**
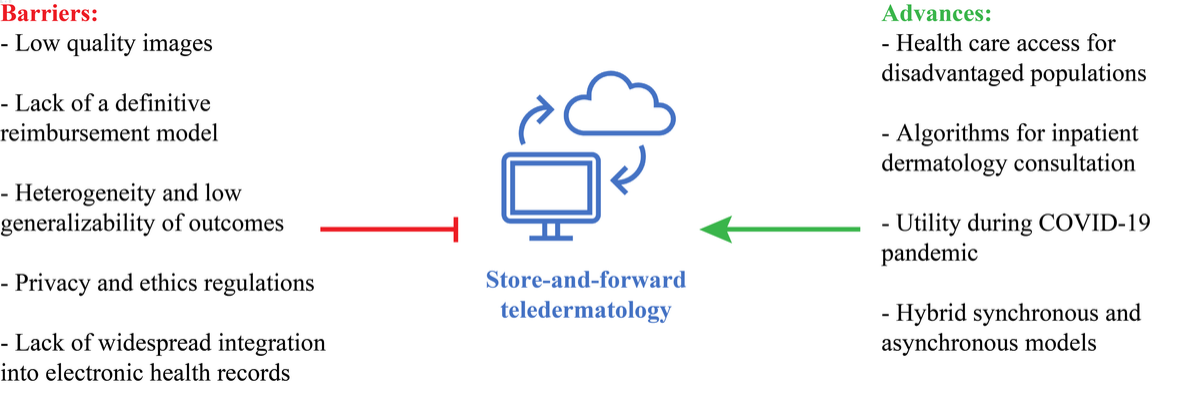
Barriers and advances to the integration of store-and-forward teledermatology into clinical practice.

One practical advantage of asynchronous teledermatology is the potential to expand health care access to underserved populations (Figure 1). Several urban programs have used images obtained during PCP visits for SAF teledermatology consultation in safety-net health care systems [26-29]. All of the studies found that asynchronous consultation resulted in substantially reduced waiting periods for dermatologic care compared to traditional referral systems [26-29]. One study in particular found that the no-show rate for referral via SAF consultation was around 60% of the no-show rate through traditional referral [29]. SAF teledermatology consultation has also been studied in rural populations, though outcomes have largely been limited to clinician questionnaires and economic analyses [30,31]. As health care systems expand access to dermatologic care and reduce waiting intervals via asynchronous consultation, other clinically relevant outcomes such as improved quality of life and prevention of disease should be reported in future studies. Excitingly, the American Academy of Dermatology has recently introduced a telemedicine program that uses SAF media from referring clinicians to provide care to underserved US populations [32].

### Impact of COVID-19

#### Outpatient Management

The COVID-19 pandemic necessitated many clinics to temporarily adopt teledermatology for all patient encounters. Although most teledermatology visits were synchronous, asynchronous visits drastically increased from prior years [33-35]. For example, one program reported 3 asynchronous visits in April 2019 and 197 asynchronous visits in April 2020, increasing from <1% of all patient encounters to approximately 10% [33,34]. Another group reported that the average number of daily teledermatology consultations received increased from 9.28 to 36.4 following an alert regarding the potential cutaneous manifestations of COVID-19 [36].

Several important considerations arose following the widespread adoption of teledermatology. For patients who communicated directly with their dermatologists, it was important to explore whether the circumstances that used their self-acquired SAF images were appropriate. Das et al [37] used patient-submitted images to adjust isotretinoin dosing in established acne patients and discovered no significant difference in the dosing regimens between synchronous and asynchronous visits. A group in Spain used a direct-to-patient teledermatology mobile app to evaluate new patients who submitted their own photographs [38]. Since the most common conditions they encountered were nevi, acne, and eczema, they were able to delay in-person visits for at least 3 months in 85% of their cohort, although the long-term outcomes of postponing these appointments are unknown [38]. Kazi et al [39] found that immunomodulatory and biologic therapies were more frequently prescribed with synchronous encounters, whereas antibiotics and nonretinoid acne medications were more frequently prescribed with asynchronous encounters using patient-generated photographs. This may indicate that SAF teledermatology is less appropriate for the management of complex medical dermatology than synchronous teledermatology [39]. Current data suggest that patient-submitted images are useful for managing well-established, straightforward conditions such as acne. However, more research is needed to investigate other highly relevant clinical outcomes, such as the quality of life and prevention of disease.

For clinicians referring patients to dermatology, additional considerations included the triage of patients based on their skin condition and the outcomes of triage. A group in England conducted a pilot study for skin cancer referrals in which patients were triaged based on clinician-taken photographs [40]. They found that 43.8% of patients were allocated a clinic appointment, 20.2% of patients were booked for dermatologic surgery, and 35.1% of patients avoided a FTF visit [40]. It is conceivable that an even larger proportion of patients could avoid FTF appointments for general dermatologic concerns. For instance, Bergamo et al [41] observed that 84% of teledermatology consultations from PCPs involved diagnostic and therapeutic recommendations that avoided FTF visits [41]. Similar to research involving patient-generated images, data on the clinical outcomes of postponing or avoiding dermatology clinic visits are needed.

#### Inpatient Management

Unlike the outpatient setting, research on SAF teledermatology in the inpatient setting is limited to clinician-initiated images. Prior to the COVID-19 pandemic, data regarding asynchronous teledermatology for inpatient consultations were scarce. Barbieri et al [42] found that SAF teledermatology was potentially useful for triaging inpatient consultation, as teledermatologists agreed with in-person dermatologists on the need for same-day evaluation and biopsy in >90% of consultations [42].

During the COVID-19 pandemic, the integration of asynchronous teledermatology into inpatient consultations substantially increased as dermatology departments sought to maximize patient safety by minimizing unnecessary clinical exposures [35]. Consequently, some medical centers developed triage algorithms using SAF images to minimize physical contact [35,43,44]. The value of asynchronous teledermatology versus in-person evaluation for inpatient consultation depends on the medical decision in question. For instance, studies reported agreement ranging from 66% to 74% in the need to obtain a biopsy and diagnostic agreement ranging from 56% to 66.7% between teledermatologists and in-person dermatologists [42,45,46]. Gabel et al [46] observed near-perfect agreement in treatment decision but almost no agreement in next-day planning, which consisted of variations of outpatient follow-up and signing off versus continued inpatient monitoring. Keller et al [45] found that web-based and in-person dermatology consultations resulted in similar rates of change in diagnoses and treatment compared to initial decisions made by the primary team. However, agreement in the diagnosis itself (45.3%), systemic therapy (52.8%), and need for obtaining a biopsy (66%) were somewhat discordant, indicating that these changes in decision-making may yield different clinical outcomes [45]. In addition to the dearth of research, a major limitation of the literature on SAF teledermatology for inpatient consultations is the heterogenous measures of medical decision-making reported across different studies. Therefore, meta-analyses that examine interobserver agreement for discrete medical decisions, such as decision to biopsy or the initiation of systemic therapy, are needed.

## Discussion

### Principal Findings

SAF teledermatology uses electronically stored information, including patient photographs and demographic information, for clinical decision-making asynchronous to the patient encounter. The integration of SAF teledermatology into clinical practice has been increasing in recent years, especially during the COVID-19 pandemic. This narrative literature review explored 47 articles by a key element of study design—whether the images were acquired by a trained clinician or the patient, as the quality and utility of the images may vary by the clinical expertise of the photographer. In general, photographs taken by trained clinicians rather than patients are higher quality and have better and more relevant diagnostic and clinical outcomes. SAF teledermatology helped clinicians avoid unnecessary physical contact with patients in the outpatient and inpatient settings during the COVID-19 pandemic.

### Future Directions

The growth and increased use of SAF teledermatology following the COVID-19 pandemic is evident. However, it remains unclear how SAF teledermatology will continue to be integrated into dermatologic practice. A cross-sectional study surveying the Association of Professors of Dermatology observed that most respondents (89%, 31/35) found the implementation of SAF images alongside video or phone calls the most feasible for teledermatology visits [47]. Of those who were most ready for teledermatology implementation, all respondents indicated they would continue to use teledermatology after the pandemic [47]. Havele et al [48] reviewed 1110 pediatric dermatology video visits and 89 SAF consultations with surveys embedded into every web-based encounter. Most respondents (76%) used parent-submitted photographs to supplement video visits, and a majority (73.4%) of clinicians who lacked photographs believe that photographs would have helped with the diagnosis [48]. Therefore, hybrid teledermatology visits using both synchronous and asynchronous communication may become more prevalent in practice [49].

### Barriers to Implementation

Substantial barriers must be overcome before SAF teledermatology can be implemented into standard dermatologic care across multiple systems of practice (Figure 1). Adherence to established privacy and ethics regulations may pose substantial medicolegal risks to clinicians capturing patient photographs [50]. For this reason, clinicians should obtain proper patient consent, explain how images will be used, and delete the images from their smartphones after being uploaded to patient charts while ensuring sufficient security in their digital communications [50]. In general, patients prefer giving verbal consent and their photographs being taken by clinic- or hospital-owned cameras [51]. EHR programs such as Epic and Cerner as well as new mobile apps allow for the secure upload of patient images to their medical charts without permanent storage on the user’s device [52].

Secure apps that combine SAF images with patient communication could streamline the delivery of teledermatology care. Such apps currently exist but may be difficult to use, lack EHR integration, or incur substantial out-of-pocket costs to patients [53]. Kim et al [26] developed a SAF teledermatology consultation workflow built within an Epic-based EHR, which could simplify asynchronous dermatology consultation, especially for large health care networks with a unified EHR.

Furthermore, increased clinician workload and the lack of a definitive reimbursement model cause asynchronous teledermatology to be a substantial burden or gamble for many practices [54-56]. Currently, Medicaid reimburses clinician-initiated SAF teledermatology consultation in fewer than half of all US states, whereas Medicare only reimburses as part of telemedicine demonstration programs in Alaska and Hawaii [57]. Reimbursement for the evaluation of patient-submitted images has been proposed but not implemented by the Centers for Medicare and Medicaid Services [56]. Given that telephone-based consultation has a definitive reimbursement model that has become more flexible following the pandemic, a similar policy should be considered for SAF teledermatology services, especially those that supplement other web-based appointments [49]. Patient privacy, complex SAF teledermatology workflows, and the lack of a definitive reimbursement model are key challenges that need to be addressed with more widespread adoption of SAF teledermatology.

### Limitations

This narrative literature review was limited by the sole inclusion of studies published in English that were available in PubMed and Google Scholar, which may have excluded other important studies not available in English or not indexed in these databases. Our review included both qualitative and quantitative studies; although both study types are valuable for learning about SAF teledermatology, quantitative outcomes may be more relevant and prognostic for health care systems considering the implementation of new SAF programs. Furthermore, many prospective studies included in this review involved motivated patient cohorts or referring clinicians who were equipped with thorough instructions. These conditions are often not representative of actual clinical practice and could have limited applicability to a real-life setting. Finally, many studies included in this review used patient cohorts with relatively small sample sizes (<100 subjects) and consequently reported descriptive outcomes or had wide variability in their data. More quantitative studies on the outcomes of SAF teledermatology with larger cohorts are needed.

### Conclusion

SAF teledermatology has a growing role in dermatology with increasingly promising diagnostic utility and clinical outcomes over the past 2 decades. Assessing SAF teledermatology by whether images are submitted by patients or clinicians can illuminate key differences in outcomes. For instance, image quality and diagnostic concordance are generally lower and more variable with patient-submitted images, which may impact their decision-making utility. SAF teledermatology can improve the efficiency of and access to care when photographs are taken by either clinicians or patients. Only the long-term clinical outcomes of clinician-submitted images have been studied, albeit to a limited extent. Amid the COVID-19 pandemic, the use and role of SAF teledermatology rapidly expanded in the inpatient and outpatient settings. For the outpatient setting, asynchronous teledermatology helped avoid FTF visits unless necessary, as many uncomplicated conditions could be managed remotely via images captured by patients and referring clinicians. For the inpatient setting, SAF teledermatology minimized unnecessary contact during dermatology consultations, although current studies are limited by the heterogeneity of their outcomes. Asynchronous teledermatology will likely play a greater role in the future, becoming incorporated into hybrid SAF and video teledermatology models. However, the obstacles summarized in this review should be addressed before its widespread implementation into clinical practice.

## Abbreviations

EHR: electronic health record
FTF: face-to-face
PCP: primary care physician
SAF: store-and-forward
